# The GDF‐5 mutant M1673 exerts robust anabolic and anti‐catabolic effects in chondrocytes

**DOI:** 10.1111/jcmm.15149

**Published:** 2020-06-04

**Authors:** Tanja Mang, Kerstin Kleinschmidt‐Dörr, Frank Ploeger, Sven Lindemann, Anne Gigout

**Affiliations:** ^1^ Osteoarthritis Research Merck KGaA Darmstadt Germany; ^2^ Institute for Organic Chemistry and Biochemistry Technische Universität Darmstadt Germany; ^3^ Biopharm GmbH Heildelberg Germany

**Keywords:** cartilage, chondrocytes, disease‐modifying osteoarthritis drug, growth and differentiation factor 5, osteoarthritis

## Abstract

The growth and differentiation factor 5 (GDF‐5) is known to play a key role in cartilage morphogenesis and homeostasis, and a single‐nucleotide polymorphism in its promoter sequence was found to be associated with osteoarthritis (OA). In addition, GDF‐5 was shown to promote extracellular matrix (ECM) production in healthy chondrocytes, to stimulate chondrogenesis of mesenchymal stem cells (MSCs) and to protect against OA progression in vivo. Therefore, GDF‐5 appears to be a promising treatment for osteoarthritis. However, GDF‐5 also promotes osteogenesis and hypertrophy, limiting its therapeutic utility. To circumvent this, a GDF‐5 mutant with lower hypertrophic and osteogenic properties was engineered: M1673. The present study aimed to evaluate and compare the effects of GDF‐5 and M1673 on primary porcine and human OA chondrocytes. We found that both GDF‐5 and M1673 can robustly stimulate ECM accumulation, type II collagen and aggrecan expression in porcine and human OA chondrocytes in 3D culture. In addition, both molecules also down‐regulated MMP13 and ADAMTS5 expression. These results suggest that M1673 retained the anabolic and anti‐catabolic effects of GDF‐5 on chondrocytes and is an alternative to GDF‐5 for osteoarthritis.

## 1 INTRODUCTION

Osteoarthritis (OA) is the most common joint disease worldwide and an important cause of disability for an increasing number of patients.^1^ One of the characteristics of the disease is the progressive degradation of articular cartilage due to an excess of catabolic over anabolic activities. Because cartilage has only a limited self‐repair capacity, only a therapeutic intervention could possibly stimulate tissue repair and restore the articular surface.^2^ However, the currently available therapies focus on pain relief only and there is no treatment option to stop, slow down or reverse cartilage degradation. Consequently, there remains a strong, unmet medical need for disease‐modifying osteoarthritis drugs (DMOADs), which can be pro‐anabolic (stimulating cartilage regeneration), anti‐catabolic (inhibiting further tissue destruction) or which can combine both properties.^3^

The growth and differentiation factor 5 (GDF‐5) is an endogenous growth factor, which plays an important role during the embryonic formation of articular cartilage and for its maintenance during adulthood.^4^ GDF‐5 has been shown to increase matrix production in bovine^5^ or healthy human chondrocytes^6^ and to decrease the expression of the proteases MMP13 and ADAMTS4 in healthy human chondrocytes.^7^ In a fracture healing model in rabbits, GDF‐5 prevented calcified callus fusion and caused neoformation of cartilage, fulfilling histological criteria of hyaline joint cartilage.^8^ GDF‐5 was also demonstrated to stimulate chondrogenesis of mesenchymal stem cells (MSC)^9^, ^10^, ^11^, ^12^ and to ameliorate osteoarthritis in a chronic OA rat model.^13^ In addition, a single‐nucleotide polymorphism (SNP) in the promoter sequence of the GDF‐5 gene leading to a reduced GDF‐5 expression^14^, ^15^ was found to be associated with OA in a genome‐wide association study.^16^ Taken together, these results indicate that GDF‐5 plays a key role in cartilage morphogenesis and homeostasis and presents a great therapeutic potential for cartilage repair. However, GDF‐5 also stimulates hypertrophy in MSCs^10^ and osteophyte formation in a rat model of OA.^12^ These properties might limit its therapeutic utility.

To reduce the hypertrophic and osteogenic properties of GDF‐5, a variant of the molecule was created: M1673. M1673 presents a single point mutation in the type I receptor binding site of GDF‐5 resulting in modified receptor binding affinities compared with the wild‐type molecule GDF‐5. It was confirmed that M1673 stimulates chondrogenesis of MSCs similarly to GDF‐5 but has reduced hypertrophic and osteogenic properties.^17^ The aim of the present study was to evaluate if the anabolic effect of GDF‐5 on healthy chondrocytes observed by others is maintained in M1673 and to investigate the effect of both compounds on human OA chondrocytes. To achieve this objective, primary porcine chondrocytes and human OA chondrocytes from several donors were cultured in 3D with M1673 or GDF‐5. The effects of M1673 on cell proliferation, matrix production and the cell phenotype were evaluated and compared with those of GDF‐5.

## 2 MATERIALS AND METHODS

### 2.1 GDF‐5 and M1673

DNAs coding for the mature parts of human GDF‐5 proteins were isolated from human ROB‐C26 osteoprogenitor cells^18^ via RT‐PCR technique and subsequently ligated into prokaryotic plasmid vectors. The single mutations were introduced into these sequences via site‐directed mutagenesis, and proteins were expressed in E.coli, isolated from inclusion bodies, renatured and purified, as described elsewhere.^19^ The GDF‐5 mutants used in this study correspond to the previously described R399E mutant,^19^ and its drug candidate denomination is M1673. Both proteins were stored in 10 mM HCl at −80°C.

In this study, GDF‐5 and M1673 were used at 300 ng/mL, based on previous experiments which showed that the maximum effect on chondrocytes was reached at this concentration.

### 2.2 Culture medium

The culture medium was DMEM high glucose (Gibco) supplemented with 10% foetal bovine serum (Biochrom), 50 µg/mL ascorbate‐2‐phosphate (Sigma) and 400 µM proline (Sigma). Based on former experiments,^20^ the culture medium for human OA chondrocytes was adjusted to an osmolarity of 380 mOsm (373‐386 mOsm) by using sterile NaCl solution (250 mg/mL, Merck). The osmolarity was verified with a cryoscopic osmometer Osmomat 030‐3P (Gonotec).

### 2.3 Porcine chondrocyte culture

Porcine chondrocytes were isolated from the femoral heads of 1‐year‐old pigs provided by a local slaughterhouse. The cartilage was initially digested with collagenase 0.25% (collagenase NBG4, Serva) for 45 minutes and subsequently overnight with collagenase 0.1%. The resulting cell suspension was filtered and washed, and one million cells/well were incubated in a low‐binding 96‐well plate in the culture medium supplemented with 1% penicillin/streptomycin (PAN biotech). The cell constructs were then transferred into a 24‐well plates (Falcon) containing one mL of the culture medium supplemented with 300 ng/mL M1673, 300 ng/mL GDF‐5 or 12.5 µM HCL (control). The media were renewed twice a week. After a total culture period of 4 weeks, the 3D constructs were either used for gene expression (real‐time PCR, N = 3‐4) or histological analysis (N = 3) or were processed for biochemical analysis (DNA, GAG, HPro content; N = 2‐4). To do so, the 3D constructs were digested with a papain solution at 60°C overnight. The papain solution consisted of 0.125 mg/mL papain (Merck) in 0.1 M Na_2_HPO_4_ (Merck), 0.01 M EDTA (Merck) and 5 mM L‐cysteine (Merck). A total of three animals were used.

### 2.4 Human OA chondrocyte culture

For human OA chondrocyte culture, the cells were isolated from the knee or hip joints of OA patients who underwent total joint replacement surgery. Human material was provided by the clinic for orthopaedics, traumatology and sports medicine at Elisabethenstift Darmstadt, with full ethics approval (No. FF24/2015) and written consent. Cells were isolated as described for the porcine chondrocytes. Isolated human OA chondrocytes were first cultured 7 days in monolayer at 8‐10 × 10^6^ cells per PRIMARIA™ T‐75 flasks (Corning) in culture medium supplemented with 1% penicillin/streptomycin. The cells were then harvested, 2 × 10^6^ cells were resuspended in an alginate solution (1.25% alginate from Fluka in 0.2 M HEPES from AppliChem and 1.5 M NaCl from Merck, adjusted to pH 7.4), and the cell suspension was poured drop by drop in 120 mM CaCl_2_ (Merck) containing 10 mM HEPES (AppliChem). The cell drops polymerized for 15 minutes under agitation to form alginate beads and were washed three times with a 150 mM NaCl solution. The alginate beads were first cultured for 7 days without treatment in culture medium adjusted to 380 mOsm. Subsequently, the beads were transferred into 24‐well ultra‐low‐binding plates (VWR) with 5 beads/well in one mL of culture medium at 380 mOsm supplemented with 300 ng/mL M1673, 300 ng/mL GDF‐5 or 12.5 µM HCL (control). The media were renewed twice a week. After 14 days, the alginate beads were dissolved for one hour in 460 µL of 55 mM Na‐citrate (Merck) with 150 mM NaCl at pH 8 and 40 µL of 2.5% collagenase. Next, 500 µL of DMEM high glucose or PBS was added and the solution centrifuged: the cell pellet was used to evaluate the cell concentration (N = 2‐4 per donor) or gene expression (real‐time PCR, N = 3‐6 per donor). GAG (N = 6‐8 per donor) and HPro (N = 6 per donor) were measured in the dissolved alginate supernatant. A total of nine donors were used. Depending on the number of cells that could be harvested from one donor, all analyses could be performed or only some of them.

### 2.5 Cell concentration

Cell concentration in the porcine 3D cell constructs was indirectly determined by measuring the dsDNA content in the papain lysate. The Quant‐iT PicoGreen dsDNA assay kit (Invitrogen) was used according to the manufacturer instructions. The fluorescence at 485/535 nm was measured in the samples and compared with that of a lambda DNA standard (31.25‐1000 ng/mL, diluted in 1xTE buffer provided in the kit) provided in the PicoGreen kit. It has been reported that chondrocytes contain 7.7 ± 0.5 pg DNA.^21^ Consequently, the number of cells/3D construct was calculated using the formula: $\text{Million}\_\text{cell}/\text{construct}=\frac{\text{DNA}\;(\text{ng}/\text{construct})}{7.7\times 1000}$

For human cells cultured in alginate, the cell concentration was determined by using the automated trypan blue dye exclusion method with a Vicell XR analyser (Beckman Coulter).

### 2.6 Glycosaminoglycan (GAG) analysis

A dimethylmethylene blue (DMMB) assay was used to quantify GAG in the samples. The absorbance at 540/595 nm was compared with that of chondroitin sulphate standards (0.78‐50 µg/mL, diluted in PBS; Sigma).

### 2.7 Hydroxyproline (HPro) analysis

The HPro concentration was determined by using high‐performance liquid chromatography‐mass spectrometry/mass spectrometry (HPLC‐MS/MS) assays. 4‐hydroxyproline (0.1‐200 µg/mL, diluted in medium; VWR) was used as calibration standard. A mixture of 5 µL sample, 10 µL 4‐hydroxyproline (1.2 µg/mL) and 200 µL HCL (25%) was hydrolysed overnight at 110°C. The mixture was centrifuged, evaporated at 55°C/10 Torr, resuspended in 1 mL MilliQ‐water and mixed 1/5 in acetonitrile (Merck) for injection into the HPLC‐MS/MS system. High‐performance liquid chromatography separation was performed on a hydrophilic interaction chromatography (HILIC) column (Sequant ZIC‐HILIC, 50‐2.1 mM, 3.5 µM, 200 Å column; Merck) with a mobile phase gradient (eluent A: 0.1% formic acid from Merck, eluent B: acetonitrile from Merck). Detection was performed with a Tandem MS (API4000, Sciex) with a turbo ion spray interface operating in positive ion mode.

### 2.8 Quantitative real‐time PCR (qRT‐PCR)

Cell lysis and ribonucleic acid (RNA) isolation was performed with the RNeasy Mini Kit (Qiagen). Cells were homogenized in RLT buffer, treated with proteinase K, and the RNA was extracted according to the manufacturer instructions. The concentration and quality of the isolated RNA were analysed by capillary gel electrophoresis using an Agilent 600 Nano Chip with an Agilent 2100 Bioanalyzer. Afterwards, a reverse transcription of the isolated RNA was realized with the SuperScript III First‐Stranded Synthesis Supermix Kit (Invitrogen). qRT‐PCR was performed with the SYBR‐Green Jumpstart Taq Ready Mix (Sigma) by using 200 nM of the forward and reverse primer (from Eurofins MWG Operon, see Table 1) and was run in the thermocycler Mx300P (Agilent). For each sample, the PCR reaction was performed in duplicates against the housekeeping gene (HKG; EF1α for human samples, RPL13A for porcine samples). For each gene of interest (GOI), the cycle threshold (Ct) was analysed and the relative abundance calculated with the formula $\text{relative abundance}=2^{\left(C_{T}\text{HKG}-C_{T}\text{GOI}\right)}$ .

**Table 1:** PCR primers

Porcine RPL13A	Forward: 5′‐TCAAGGTGGTGCGTCTGAAG‐3′ Reverse: 5′‐TACGTTCTTTTCCGCCTGCT‐3′
Porcine Aggrecan	Forward: 5′‐GCTTATGCCTTCCCAGCTAC‐3′ Reverse: 5′‐GATGCTGCTCAGGTGTGACT‐3′
Porcine Collagen I	Forward: 5′‐CCCCAGAAGAACTGGTACAT‐3′ Reverse: 5′‐CCTACAGGTACCCTGTGTCC‐3′
Porcine Collagen II	Forward: 5′‐GGATGGGCAGAGGTATAATG‐3′ Reverse: 5′‐TCTCCAGGTTCTCCTTTCTG‐3′
Bovine EF1α	Forward: 5′‐AGCTGAAGGAGAAGATTGATC‐3′ Reverse: 5′‐GGCAGACTTGGTGACCTTG‐3′
Bovine Aggrecan	Forward: 5′‐GAAACCTCTGGACTCTTTGGTGTC‐3′ Reverse: 5′‐GCCAGATATTTCTCCATAAAACCCTGA‐3′
Bovine Collagen I	Forward: 5′‐TGGCCCAGAAGAACTGGT‐3′ Reverse: 5′‐AGGAAGGTCAGCTGGATG‐3′
Bovine Collagen II	Forward: 5′‐GAACCCAGAACCAACACAATCC‐3′ Reverse: 5′‐TCTGCCCAGTTCAGGTCTCTTAGAGA‐3′
Human EF1α	Forward: 5′‐CCTTGTGGAAATTTGAGACC‐3′ Reverse: 5′‐CCATTTTGTTAACACCGACA‐3′
Human ADAMTS5	Forward: 5′‐TCAAAGCCAAAGACCAGACT‐3′ Reverse: 5′‐ATTTCCTTCGTGGCAGAGTA‐3′
Human Aggrecan	Forward: 5′‐GAAAGGCATCGTGTTCCATT‐3′ Reverse: 5′‐ACGTCCTCACACCAGGAAAC‐3′
Human Collagen I	Forward: 5′‐AAAGGATCTCCTGGTGAAGC‐3′ Reverse: 5′‐CACCTTTAGGTCCAGGGAAT‐3′
Human Collagen II	Forward: 5′‐CCTGAGTGGAAGAGTGGAGA‐3′ Reverse: 5′‐TCCATAGCTGAAATGGAAGC‐3′
Human MMP13	Forward: 5′‐CCAACCCTAAACATCCAAAAAC‐3′ Reverse: 5′‐AAAAACAGCTCCGCATCAAC‐3′

### 2.9 Histology

Porcine 3D cell constructs were fixed with 4% paraformaldehyde (VWR), dehydrated with an increasing alcohol series and infiltrated with, as well as embedded in, paraffin (Merck). Cell samples were cut with the rotary microtome RM2255 (Sigma) in 5 µM slices, which were subsequently dried on a heat plate and analysed for different extracellular matrix molecules. For the histochemical staining, the slices were stained with 0.025% Safranin O (Sigma) and 0.1% Fast Green (Sigma) as a counter stain by using the multistainer ST5020 (Leica). For immunohistochemical detection of type I and II collagens, the immunohistochemistry stainer Leica Bond III, combined with the polymer refined detection kit from Leica (type I collagen) or the anti‐rabbit HRP Kit from DAKO (type II collagen), was used. Type I collagen was detected by using a mouse anti‐collagen I antibody (1:200, Abcam), and type II collagen was detected by using a rabbit anti‐collagen II antibody (2 µg/mL, Abcam). The slices were digitalized by using the Leica Scanner SCN400.

### 2.10 Statistics

Statistics were analysed with the software GraphPad Prism v7.00. One‐way analysis of variance (ANOVA) or repeated‐measures ANOVA was used to analyse intra‐ or inter‐animal/donor differences, respectively. No correction for multiple comparisons was conducted in order to limit type II errors. For the human data, outliers were identified with the ROUT method and excluded from the graph presenting all donors together and from the statistical analysis.

## 3 RESULTS

### 3.1 GDF‐5 and M1673 stimulate cell proliferation and extracellular matrix molecule production in porcine chondrocyte 3D culture

The effect of GDF‐5 and M1673 was analysed in porcine chondrocyte 3D culture. Three experiments were realized with chondrocytes harvested from three different animals. The cells were cultured in suspension enabling them to spontaneously aggregate to form a cell/matrix construct. After 4 weeks of culture without treatment or in the presence of GDF‐5 or M1673 at 300 ng/mL, the cell content reflecting cell proliferation was analysed, together with the GAG and the HPro content reflecting proteoglycan and collagen production, respectively. The expressions of aggrecan, type I and type II collagen were also evaluated, and a histological staining of the constructs was performed.

**Figure 1:**
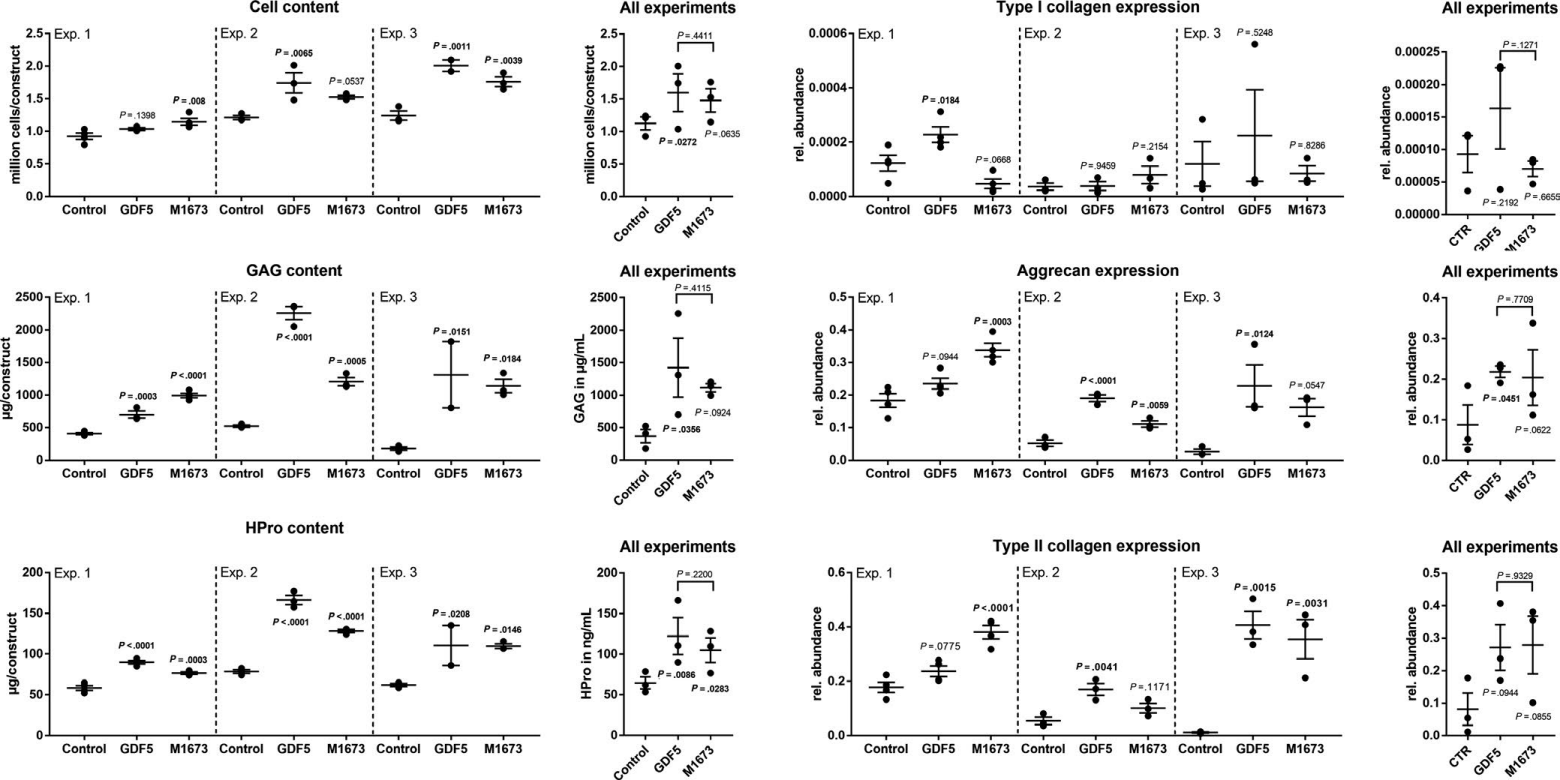
Porcine chondrocytes were cultured in 3D with GDF‐5 or M1673 at 300 ng/mL or left untreated (control). After 4 wk, the cell, GAG and HPro contents, as well as the expression of type I, type II collagen and aggrecan, were analysed. Three experiments were conducted with cells from three different animals. The results for each experiment separately (with n = 2‐4 biological replicates) and all experiments together (with one point being the average of the biological replicates obtained from one animal, N = 3) are shown to illustrate both the inter‐ and intra‐animal variability. *P*‐values are shown in bold when <.05

Both GDF‐5 and M1673 significantly increased the cell content in two of the three experiments and significantly increased GAG and HPro in all three experiments (Figure 1). In accordance with these results, aggrecan and type II collagen expression were also significantly elevated in the presence of GDF‐5 and M1673 in two of the three experiments (Figure 1). No consistent impact of GDF‐5 or M1673 could be observed on type I collagen expression; this was elevated in presence of GDF‐5 in the first experiment, but not in the other two experiments.

Overall statistical analysis was conducted on the data from the three experiments to evaluate the robustness of the inter‐animal observed effects. GDF‐5 was found to have a significant effect on the cell, GAG and HPro contents, as well as on aggrecan expression, while M1673 showed a significant effect only for the HPro content. The low number of experiments, the inter‐animal variability and a slightly smaller effect of M1673 compared with GDF‐5 can explain why M1673 did not reach significance on other readouts.

**Figure 2:**
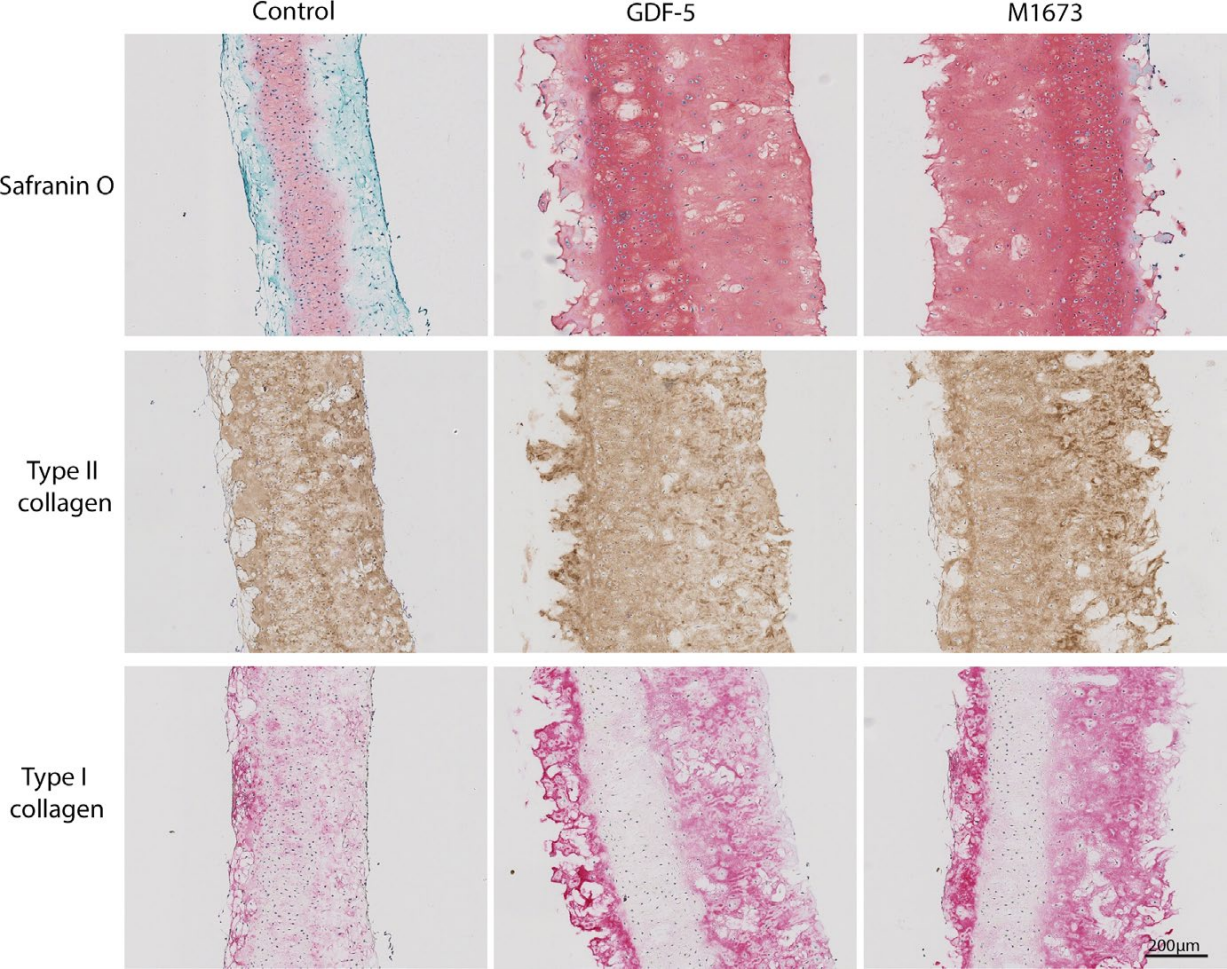
Histological analysis of porcine chondrocytes cultured in 3D with GDF‐5 or M1673 at 300 ng/mL or left untreated (control). The constructs were stained with Safranin O or for type II and type I collagen and counterstained with Fast green

Representative results from the histological staining of the 3D cell constructs are shown (Figure 2). The constructs treated with GDF‐5 and M1673 were larger than the untreated constructs and were strongly stained for Safranin O through their whole width. All constructs were strongly stained for type II collagen. Type I collagen staining was positive in the treated and untreated constructs, but, interestingly, the staining distribution was different; it appeared to be uniformly distributed in the untreated construct but located in the outer third of the constructs in GDF‐5 and M1673 treated samples. The reason for this is unclear.

### 3.2 GDF‐5 and M1673 stimulate extracellular matrix molecule production and repress protease expression in human OA chondrocytes in 3D culture

The effects of GDF‐5 and M1673 were subsequently evaluated on human chondrocytes derived from several OA patients. For each measured parameter, a minimum of five donors were tested. In contrast with porcine cells, human OA chondrocytes are difficult to culture in a scaffold‐free 3D system. Human OA chondrocytes do not produce as much ECM as healthy chondrocytes and tend not to aggregate into cell/matrix constructs. For this reason, alginate culture was used.

**Figure 3:**
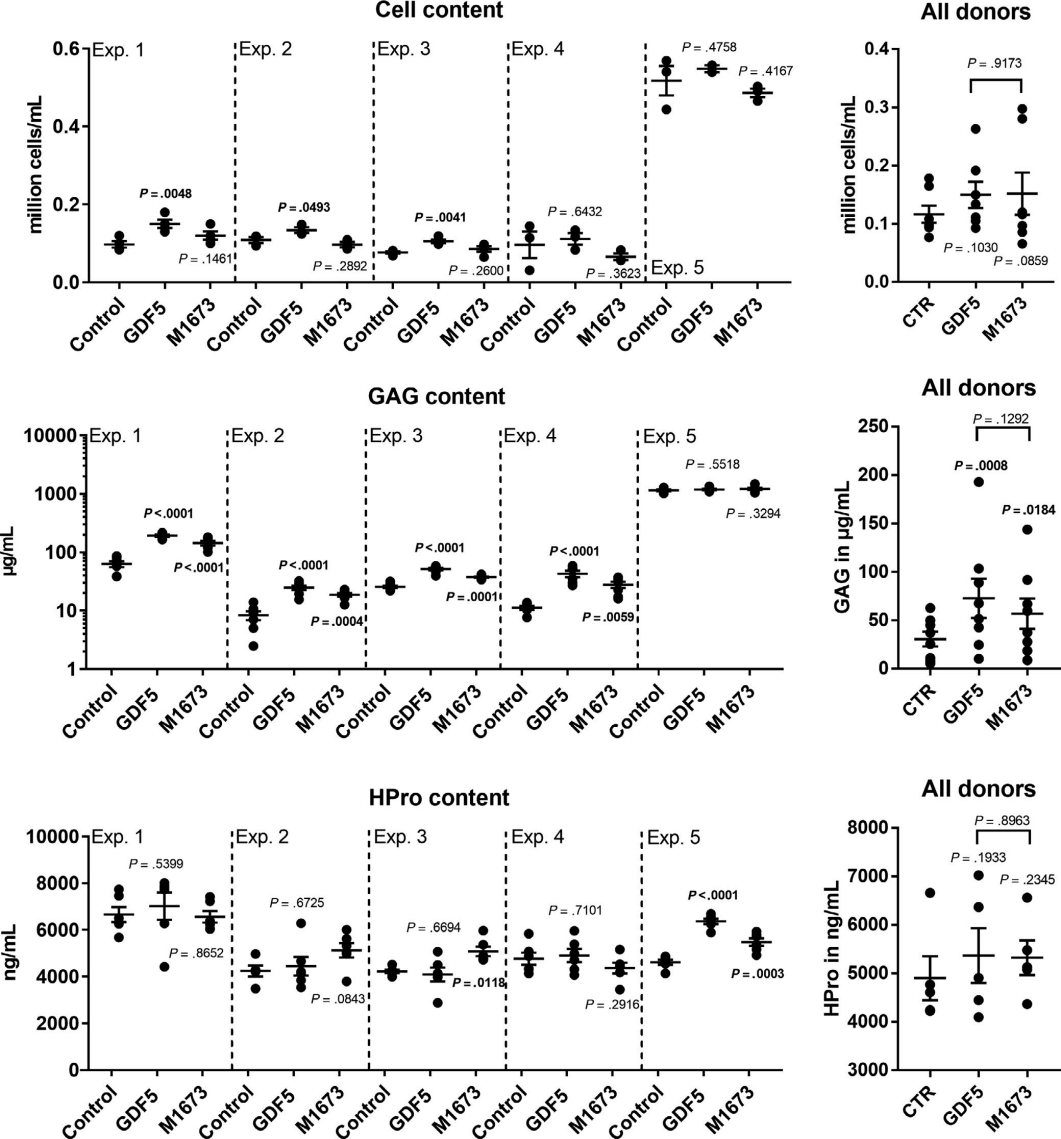
Human OA chondrocytes in alginate culture were treated with GDF‐5 or M1673 at 300 ng/mL or left untreated (control). After 2 wk, the cell, GAG and HPro contents were evaluated in the alginate. The results of five representative experiments carried out with cells from five different donors are shown separately (with n = 2‐8 biological replicates), as well as for all tested donors together (with one point being the average of the biological replicates obtained from one donor, N = 5‐8), to illustrate both the inter‐ and intra‐donor variability. *P*‐values are shown in bold when <.05

The cell, GAG and HPro contents in the alginate were analysed (Figure 3). The cell content was not influenced by M1673 but was slightly increased by GDF‐5 in three of the five donors. The GAG content was higher with both compounds in four donors while no effect could be observed in the fifth donor. Interestingly, in this fifth donor, the cell and the GAG content were much higher compared with the four other donors. It is possible that the already high GAG content in the untreated cells prevented further induction by GDF‐5 and M1673. On the contrary, the HPro content was increased by both GDF‐5 and M1673 in the fifth donor but not in the other four cases. Similarly to the porcine chondrocyte culture, all the results from several donors were analysed together to evaluate the inter‐donor reproducibility. GDF‐5 and M1673 were found to significantly influence the GAG release.

**Figure 4:**
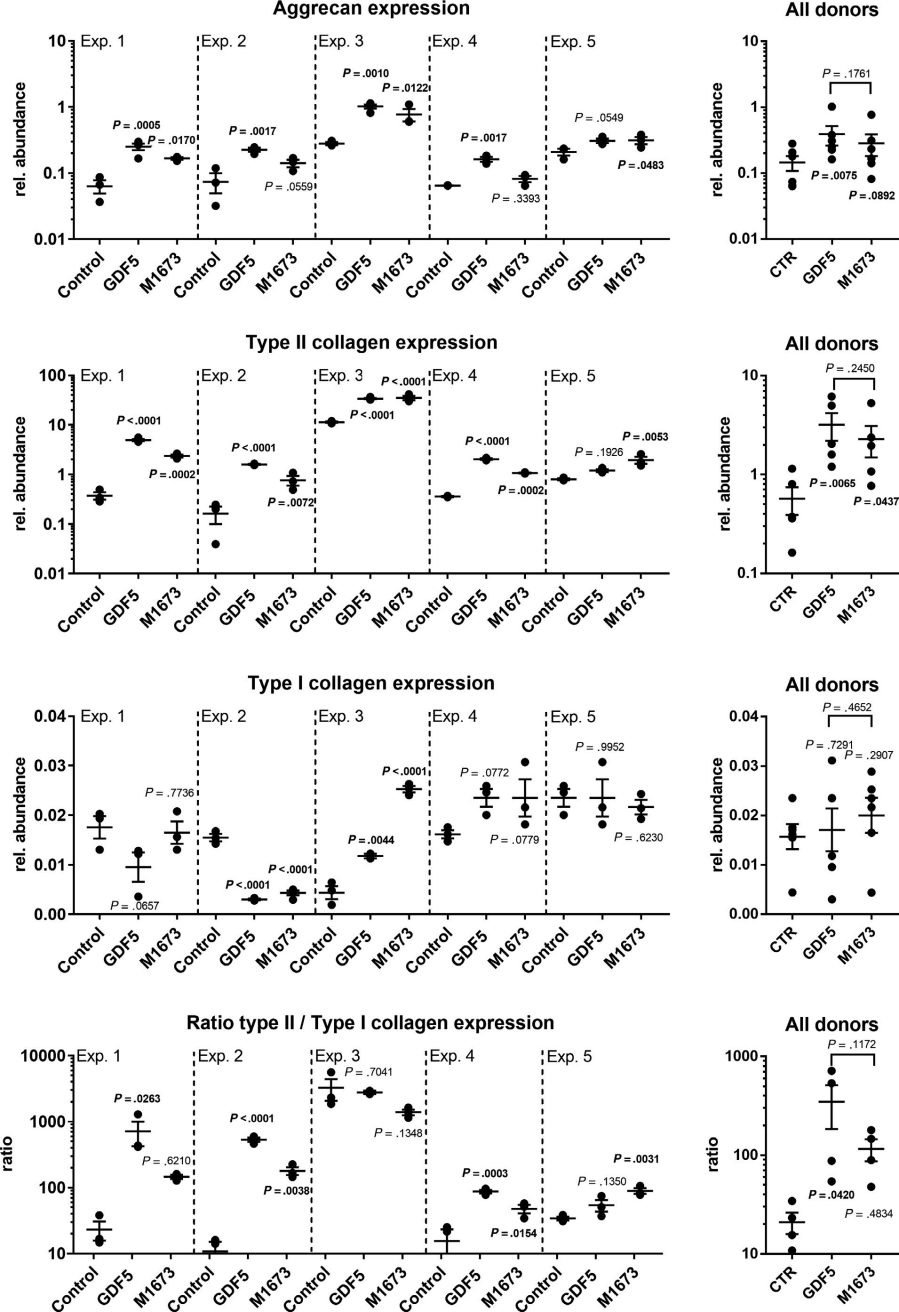
Human OA chondrocytes in alginate culture were treated with GDF‐5 or M1673 at 300 ng/mL or left untreated (control). After 2 wk, the expression of aggrecan and type II and I collagen was evaluated. The ratio of type II versus type I collagen was also calculated. The results of five representative experiments conducted with cells from five different donors are shown separately (with n = 3‐4 biological replicates), as well as for all tested donors together (with one point being the average of the biological replicates obtained from one donor, N = 5‐6), to illustrate both the inter‐ and intra‐donor variability. *P*‐values are shown in bold when <.05

Extracellular matrix molecules analysis (Figure 4) revealed that aggrecan expression was increased in all donors with GDF‐5 and in three donors with M1673. Similarly, type II collagen expression was increased in four donors with GDF‐5 and in all five donors with M1673. Regarding type I collagen expression, no consistent effect of GDF‐5 or M1673 was observed. Both molecules increased type I collagen in one donor, decreased it in another and had no effect in the other three donors. Finally, to evaluate the effect of GDF‐5 and M1673 on the cell phenotype, the ratio of type II collagen expression versus type I collagen expression was calculated. The ratio was increased in three donors with GDF‐5 and M1673. The analysis of all donors showed that GDF‐5 had a robust stimulating effect on aggrecan expression and both GDF‐5 and M1673 had a reproducible stimulating effect on type II collagen expression. In addition, GDF‐5 significantly increased the ratio of type II collagen expression versus type I collagen expression.

**Figure 5:**
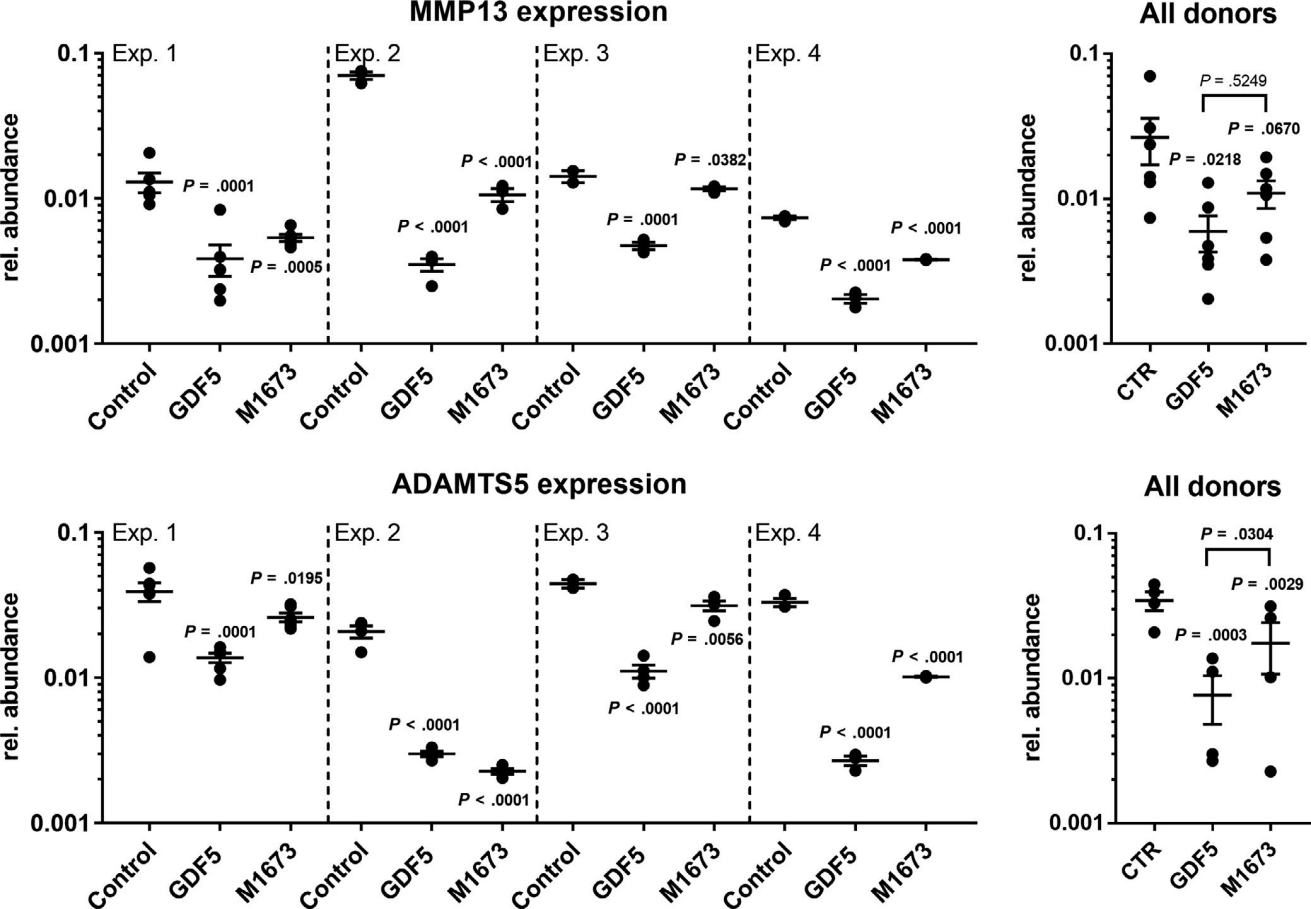
Human OA chondrocytes in alginate culture were treated with GDF‐5 or M1673 at 300 ng/mL or left untreated (control). After 2 wk, the expression of ADAMTS5 and MMP13 was evaluated. The results of four representative experiments carried out with cells from four different donors are shown separately (with n = 4‐6 biological replicates), as well as for all tested donors together (with one point being the average of the biological replicates obtained from one donor, N = 4‐6), to illustrate both the inter‐ and intra‐donor variability. *P*‐values are shown in bold when <.05

Furthermore, the expression of MMP13 and ADAMTS5 was studied (Figure 5). Both were decreased by GDF‐5 and M1673 in all donors, which resulted in a significant effect in the overall analysis.

When considering the effect of M1673 compared with GDF5, the effect of M1673 was slightly inferior to the effect of GDF5 for all readouts and in most donors. Consequently, in the analysis of all donors, M1673 did not always reach significance, even if it showed a significant effect in several donors separately. However, when directly comparing the effects of GDF‐5 and M1673, no statistical difference could be observed for any of the tested parameters, except ADAMTS5 expression.

## 4 DISCUSSION

During OA, chondrocytes present an inappropriate repair response whereby the catabolic activities exceed anabolic activities resulting in cartilage destruction. An agent reversing this imbalance would have a great therapeutic potential in OA. In the present work, we evaluated the ability of GDF‐5 and a mutant of GDF‐5, M1673, to simulate anabolic activities and reduce protease production in chondrocytes. To ensure that the observed effects were robust, two species (porcine and human) with a different disease status (healthy and OA, respectively) were used. In each case, several experiments with chondrocytes isolated from multiple animals or donors were carried out.

Firstly, in healthy porcine chondrocytes, we demonstrated that GDF‐5 increases the cell, HPro and GAG content in the 3D cell constructs. In addition, type II collagen and aggrecan expression were up‐regulated and the resulting 3D cell constructs were larger and entirely stained positive for Safranin O and type II collagen. This is in accordance with previous studies which demonstrated that GDF‐5 increases proliferation, GAG and HPro accumulation in bovine chondrocyte in 3D culture.^5^ Studies with healthy human chondrocytes in alginate or in pellet culture also revealed that GDF‐5 increases proteoglycan synthesis or GAG accumulation.^6^, ^7^ These studies were all performed in a 3D cell culture system. We could find only one report evaluating the effect of GDF‐5 on human OA chondrocytes.^22^ In this study, cells were cultured in monolayer micromass culture (at high cell density) and a discordant response to GDF‐5 on gene expression was observed. The authors concluded that chondrocytes do not respond in a predictable manner to culture with GDF‐5. Interestingly, we could not observe any robust effect of GDF‐5 in monolayer culture with porcine or human OA chondrocytes either (data not shown). Therefore, it seems that chondrocytes are only responsive to GDF‐5 in 3D culture. One reason may be the rapid loss of phenotype that chondrocytes experience in monolayer culture while they maintain their chondrocyte phenotype in 3D culture.^23^ This phenotype change may also be associated with a different response to a specific stimulus. To the best of our knowledge, the present study is the first to evaluate the effect of GDF‐5 on human OA chondrocytes in 3D culture. We found that GDF‐5 stimulates a robust increase in GAG accumulation, aggrecan and type II collagen expression in several OA donors. In addition, MMP13 and ADAMTS 5 were reproducibly down‐regulated by GDF‐5.

M1673 is a mutant of GDF‐5 with reduced affinity for BMPR1A versus GDF‐5. Similarly to GDF‐5, M1673 showed a reproducible anabolic effect on porcine and human OA chondrocytes. In porcine chondrocytes, M1673 increased the cell content (in two of the three experiments), the GAG and HPro content (in all three experiments) and aggrecan and type II collagen expression (in two of the three experiments). The histological analysis also revealed that the constructs treated with M1673 were larger than the untreated constructs. In human chondrocytes, M1673 significantly increased the GAG content (in four of five experiments), aggrecan expression (in three of five experiments) and type II collagen expression (all five experiments). In human OA cells, the effect of M1673 was often slightly inferior to the effect of GDF‐5. However, for all the anabolic readouts, no significant difference was observed between GDF‐5 and M1673 when analysing all donors together. Consequently, it can be concluded that M1673 displays similar anabolic activities to GDF‐5 on primary chondrocytes. In addition, and as is the case with GDF‐5, M1673 presented anti‐catabolic properties and decreased MMP13 and ADAMTS5 expressions (in all four experiments).

In another study conducted by our group, GDF‐5 and M1673 were also compared for their chondrogenic, hypertrophic and osteogenic potential on MSCs.^17^ The results show that both GDF‐5 and M1673 can stimulate chondrogenesis, but chondrocyte hypertrophy and osteogenesis were strongly reduced with M1673. It is now known that resident MSCs are present in the joint and participate to cartilage repair following joint injury.^24^ Similarly, these cells may contribute to restore the articular surface in OA. A promising approach would be to harness these joint‐resident MSCs by stimulating their differentiation into chondrocytes leading to subsequent cartilage ECM production. In this role, M1673 appears to be advantageous over GDF‐5 because of its reduced osteogenic and hypertrophic activity that could prevent the formation of osteophytes, as observed with GDF‐5 in vivo.^13^ Consequently, a promising therapy could consist of injecting M1673 intra‐articularly in the OA joint, where M1673 could simultaneously activate ECM production by chondrocytes, reduce protease production and activate MSCs to differentiate and contribute to repair. Because M1673 is not a naturally occurring protein, it might be immunogenic. However, in in vivo studies, no immune‐mediated adverse effects could be observed, and the immunogenicity risk of M1673 is considered to be low. Nevertheless, formation of anti‐drug antibodies should be monitored in the clinic.

This study also illustrates that modifying receptor affinities of natural proteins to fine‐tune their properties is a potential therapeutic approach. For instance, in another study, BMP‐2 residues were introduced in GDF‐5 resulting in a GDF‐5 mutant with an increased affinity for BMPR1A.^8^ This mutant (called BB‐1) displayed features of both BMP2 and GDF‐5; the angiogenic effect of GDF‐5 was retained while, in comparison with GDF‐5, BB‐1 showed enhanced osteogenic properties. Both the angiogenic and osteogenic properties of BB‐1 lead to better bone repair capabilities in a critical size bone defect models in rabbits compared with GDF‐5 or BMP2, rendering BB‐1 promising for bone regeneration therapies.

In conclusion, GDF‐5 showed reproducible anabolic and anti‐catabolic effects in porcine healthy or human OA chondrocytes. A GDF‐5 mutant showing a reduced affinity for BMPR1A was engineered: M1673. Compared with GDF‐5, M1673 showed a similar anabolic and anti‐catabolic profile on chondrocytes. GDF‐5 and M1673 were also demonstrated in another study to stimulate chondrogenesis of MSCs, but M1673 was found to be less hypertrophic and osteogenic than GDF‐5. The characteristics of M1673, which combine anabolic and anti‐catabolic effects in human OA chondrocytes, stimulation of chondrogenesis in MSCs and low hypertrophic and osteogenic properties, render M1673 a promising growth factor for cartilage repair.

## CONFLICT OF INTEREST

Tanja Mang, Kerstin Kleinschmidt‐Dörr, Sven Lindemann and Anne Gigout were all employees of Merck KGaA and Frank Ploeger of Biopharm GmbH at the time of the study.

## AUTHOR CONTRIBUTION

All authors contributed to the study conception and design. Frank Ploeger designed and produced the GDF5 mutants. Tanja Mang performed the experiments, data collection and analysis. Tanja Mang and Anne Gigout drafted the manuscript which was reviewed critically by all authors. All authors have read and approved the final submitted manuscript.

## Data Availability

The data that support the findings of this study are available from the corresponding author upon reasonable request.
